# A retrospective analysis of endocrine disease in sphingosine-1-phosphate lyase insufficiency: case series and literature review

**DOI:** 10.1530/EC-22-0250

**Published:** 2022-06-28

**Authors:** Avinaash Maharaj, Ruth Kwong, Jack Williams, Christopher Smith, Helen Storr, Ruth Krone, Debora Braslavsky, Maria Clemente, Nanik Ram, Indraneel Banerjee, Semra Çetinkaya, Federica Buonocore, Tülay Güran, John C Achermann, Louise Metherell, Rathi Prasad

**Affiliations:** 1 Centre for Endocrinology, John Vane Science Centre, Queen Mary University of London, London, UK; 2 Birmingham Children’s Hospital, Birmingham, UK; 3 Centro de Investigaciones Endocrinológicas ‘Dr. Cesar Bergadá’ (CEDIE) – CONICET – FEI – División de Endocrinología, Hospital de Niños ‘Ricardo Gutiérrez’, Buenos Aires, Argentina; 4 Paediatric Endocrinology, Growth and Development Research Unit, Vall d’Hebron Research Institute (VHIR), Hospital Vall d’Hebron, CIBERER, Instituto de Salud Carlos III, Barcelona, Spain; 5 Department of Endocrinology, The Aga Khan University Hospital, Karachi, Pakistan; 6 Department of Paediatric Endocrinology, Royal Manchester Children’s Hospital, Manchester, UK; 7 Health Sciences University, Dr. Sami Ulus Obstetrics and Gynaecology, Children’s Health and Disease Education and Research Hospital, Ankara, Turkey; 8 Genetics and Genomic Medicine Research and Teaching Department, UCL Great Ormond Street Institute of Child Health, University College London, London, UK; 9 Department of Paediatric Endocrinology and Diabetes, Marmara University, School of Medicine, Istanbul, Turkey

**Keywords:** sphingosine-1-phosphate lyase, SGPL1, sphingolipids, primary adrenal insufficiency, primary gonadal insufficiency, primary hypothyroidism

## Abstract

Sphingosine-1-phosphate lyase (SGPL1) insufficiency syndrome (SPLIS) is an autosomal recessive multi-system disorder, which mainly incorporates steroid-resistant nephrotic syndrome and primary adrenal insufficiency. Other variable endocrine manifestations are described. In this study, we aimed to comprehensively annotate the endocrinopathies associated with pathogenic *SGPL1* variants and assess for genotype–phenotype correlations by retrospectively reviewing the reports of endocrine disease within our patient cohort and all published cases in the wider literature up to February 2022. Glucocorticoid insufficiency in early childhood is the most common endocrine manifestation affecting 64% of the 50 patients reported with SPLIS, and a third of these individuals have additional mineralocorticoid deficiency. While most individuals also have nephrotic syndrome, *SGPL1* variants also account for isolated adrenal insufficiency at presentation. Primary gonadal insufficiency, manifesting with microphallus and cryptorchidism, is reported in less than one-third of affected boys, all with concomitant adrenal disease. Mild primary hypothyroidism affects approximately a third of patients. There is paucity of data on the impact of *SGPL1* deficiency on growth, and pubertal development, limited by the early and high mortality rate (approximately 50%). There is no clear genotype–phenotype correlation overall in the syndrome, with variable disease penetrance within individual kindreds. However, with regards to endocrine phenotype, the most prevalent disease variant p.R222Q (affecting 22%) is most consistently associated with isolated glucocorticoid deficiency. To conclude, SPLIS is associated with significant multiple endocrine disorders. While endocrinopathy in the syndrome generally presents in infancy, late-onset disease also occurs. Screening for these is therefore warranted both at diagnosis and through follow-up.

## Introduction

**Figure 1:**
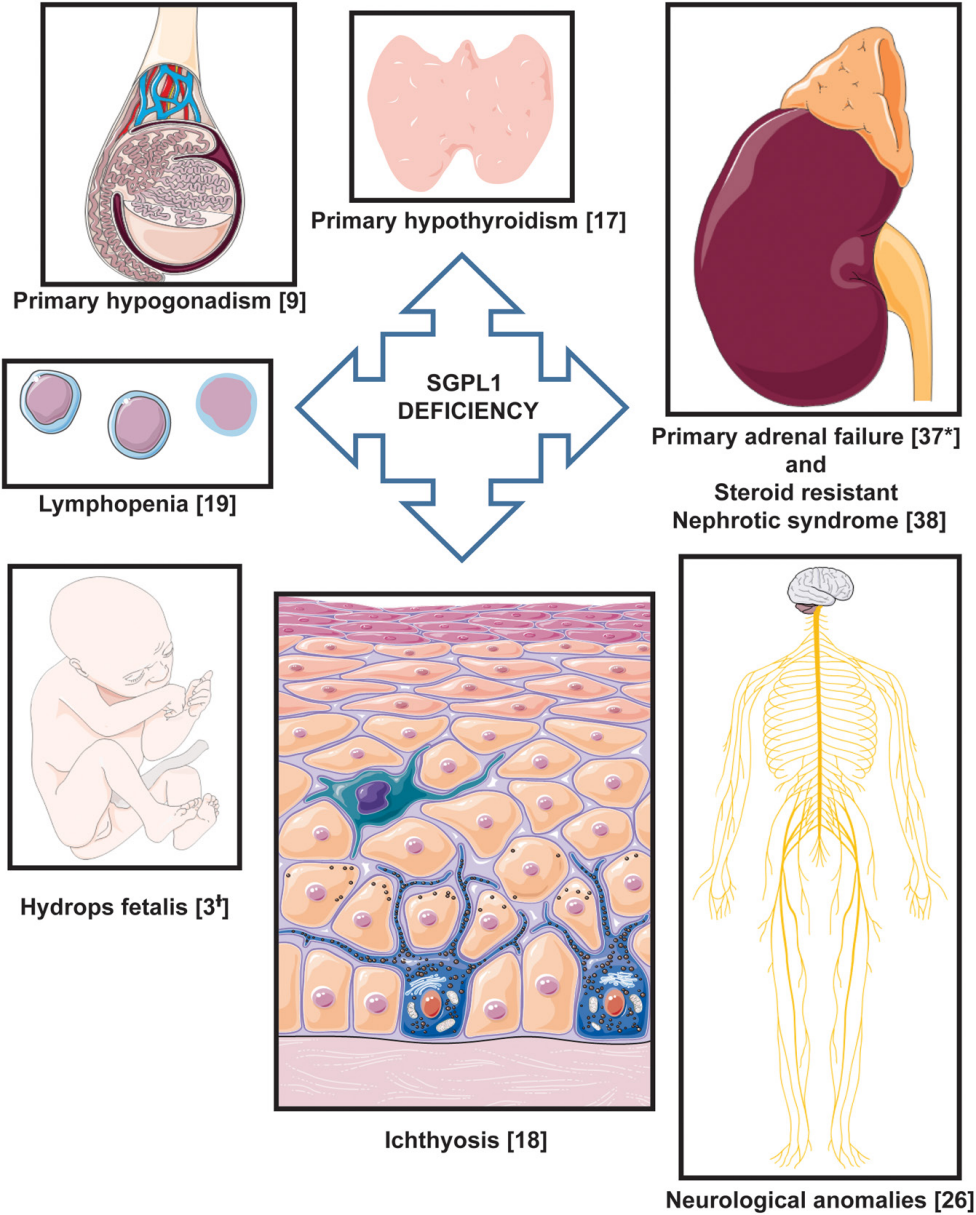
Phenotypic spectrum of human disease characterized by SGPL1 deficiency. A total of 50 patients have been reported, numbers manifesting each phenotype in parentheses. ^*^Including those with adrenal calcifications alone; ^†^Four further reports of fetal demise. This figure was created using modified images from Servier free medical art (https://smart.servier.com/).

Sphingosine-1-phosphate lyase insufficiency syndrome (SPLIS; nephrotic syndrome, type 14; NPHS14; MIM 617575), a multi-systemic disorder of sphingolipid metabolism first described in 2017, is caused by variants in *SGPL1*, in which primary adrenal insufficiency (PAI) and steroid-resistant nephrotic syndrome (SRNS) necessitating renal transplantation predominate (1, 2, 3, 4). Additional clinical features including ichthyosis, progressive neurological disease, primary hypothyroidism, primary gonadal insufficiency, lymphopenia and dyslipidaemia are reported, although not universally present in affected individuals (Fig. 1). In its most severe form, the condition results in fetal hydrops. Morbidity is significant and mortality is high, with 50% fatality, often in the first decade of childhood and most commonly associated with end-stage kidney disease and/or sepsis (2, 3, 4, 5, 6, 7, 8, 9, 10).

**Figure 2:**
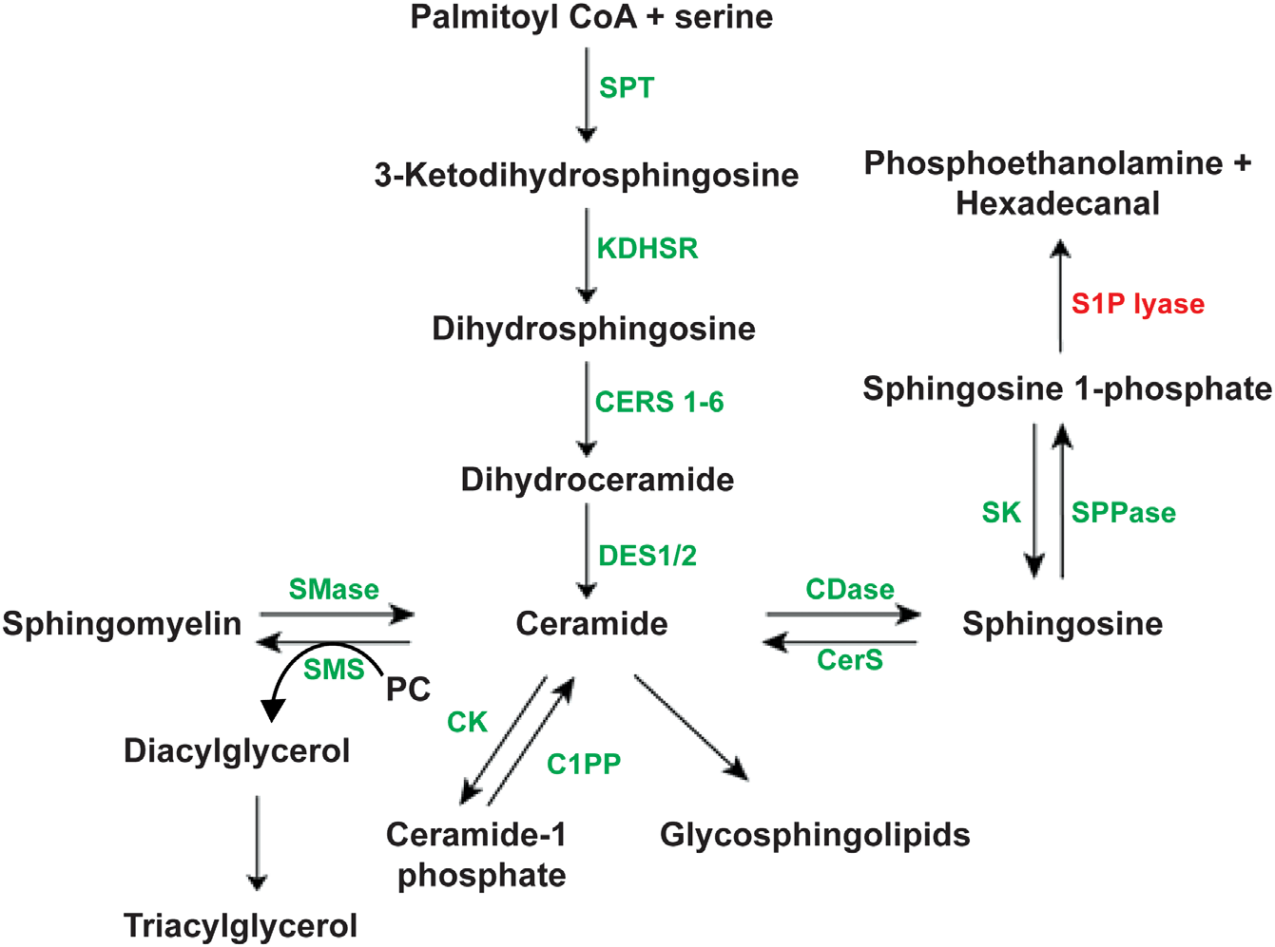
Overview of sphingolipid metabolism. Sphingolipid metabolism begins with a common entry point and exit via a single degradative pathway. SPT, serine palmitoyl transferase; KDHSR, ketodihydrosphingosine reductase; CERS, ceramide synthase; DES, dihydroceramide desaturase; SMase, sphingomylinease; SMS, sphingomyelin synthase; CK, ceramide kinase; C1PP, ceramide-1-phosphate phosphatase; CDase, ceramidase; SK, sphingosine kinase; SPPase, sphingosine-1-phosphate phosphatase; SGPL1, sphingosine-1-phosphate lyase.

Sphingolipids form structural components of mammalian plasma membranes whose roles have been implicated in signal transduction and cellular recognition pathways (11). Sphingosine-1-phosphate lyase is a ubiquitously expressed pyridoxal 5’-phosphate-dependent aldehyde-lyase responsible for the final degradative step in the sphingolipid pathway, irreversible cleavage of sphingosine-1-phosphate (S1P) and generation of 2*E*-hexadecanal and phosphoethanolamine (Fig. 2). S1P and its upstream sphingolipid intermediates, sphingosine and ceramide, are bioactive signalling molecules involved in a variety of molecular programmes and often with opposing actions (12). Mass spectrometric analysis of plasma sphingolipid intermediate levels in patients and conditioned media from patient dermal fibroblasts reveals variable elevations in upstream sphingolipids when compared to controls (3, 4). Additionally, breakdown metabolites 2*E*-hexadecanal and phosphoethanolamine are also implicated in important cellular processes including cytoskeletal reorganization, apoptosis and oxidative phosphorylation (11, 13, 14, 15). S1P lyase thus plays a critical role in the dynamicity of the sphingolipid degradative pathway, and disease related to its deficiency may be the consequence of upstream accumulation of sphingolipid intermediates in the affected tissues or indeed, deficiency of pathway products.

Since the initial descriptions of the syndrome in 2017, several case series and reports have been published, totalling 50 patients (2, 3, 4, 5, 6, 7, 8, 9, 10, 16, 17, 18, 19, 20, 21). It is clear that the endocrine phenotype within this rare disease extends beyond PAI, and in contrast to other disorders of sphingolipid metabolism, SPLIS is associated with multiple endocrinopathies. In this study, we detail the endocrine phenotypes associated with the reported genotypes within our cohort and the wider literature.

## Methods

We retrospectively reviewed clinical data for all children referred to the Centre for Endocrinology, Queen Mary University of London, who were diagnosed with bi-allelic *SGPL1* pathogenic variants (approval by the Outer North East London Research Ethics Committee, reference number 09/H0701/12), up to February 2022, totalling 13 patients. We further selected cases in the literature for inclusion in this study by querying the National Institutes of Health (NIH)/National Centre for Biotechnology Information (NCBI) PubMed electronic database on or before February 2022, for at least any of the following search terms: ‘SGPL1 mutations’; ‘SGPL1 deficiency’; ‘sphingosine-1-phosphate lyase insufficiency syndrome’; ‘SPLIS’. Three reviewers independently curated search results manually to incorporate all published clinical reports. All individuals reported with bi-allelic *SGPL1* variants were included, totalling 50 patients*.* Key endocrine clinical findings reported were assessed against SGPL1 variant, sex, age of endocrine disease presentation and ethnicity of patient. Specifically, cases (reports) were reviewed for details of adrenal disease, including absence/presence of glucocorticoid/mineralocorticoid deficiency and presence of pathology on adrenal imaging. Details of gonadal disease incorporated included clinical features (microphallus/cryptorchidism/hypospadias) or delayed puberty, biochemical findings (including gonadotrophins/sex steroids/AMH) and presence of concomitant adrenal disease. Details of thyroid disease reviewed included the presence of concomitant adrenal/nephrotic disease, diagnostic TSH and FT4 levels, and pathology identified on imaging. Where details were not given for each case, these were designated unknown (U) in the tables of results. Reports of additional endocrine manifestations were duly noted.

## Results

### Clinical phenotype of primary adrenal insufficiency in SPLIS

**Figure 3:**
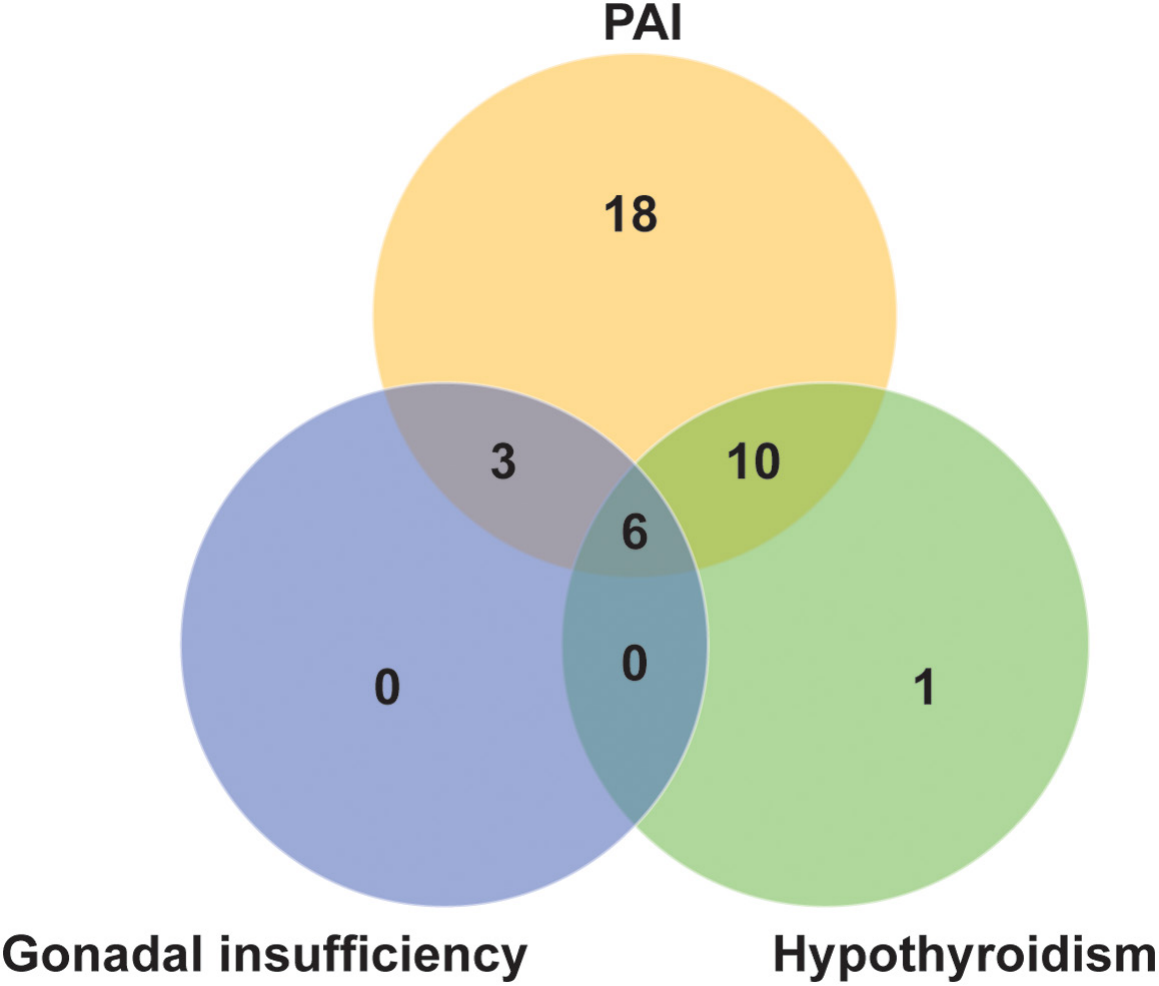
Adrenal disease, primary gonadal insufficiency and primary hypothyroidism are present variably in 38 SPLIS patients with endocrinopathy.

Endocrine dysfunction is described in 38 of the 50 patients with SPLIS (Fig. 3). PAI is the most common endocrine manifestation of the disease and indeed one of the most predominant clinical features overall. Thirty-two patients (64% of the 50 patients) presented with biochemical evidence of PAI, with a male to female preponderance of approximately 1.7:1 (overall male to female preponderance in the syndrome being 1.3:1) (Fig. 3 and Table 1) (2, 3, 4, 6, 7, 8, 9, 10, 17, 18, 19, 20, 21). The median age of PAI presentation among boys was 1 year (range, 0–6.5 years) and for girls 0.55 years (range, 0.17–11 years). Most patients presented with PAI in the first 2 years of life (81%; median age, 1 year; range, 0–11 years). Ten of the children with glucocorticoid deficiency had additional mineralocorticoid deficiency (31%) (4, 6, 7, 8, 20, 21). The majority had PAI with concomitant nephrotic syndrome, and in most cases (12 out of 27 patients), nephrotic syndrome preceded adrenal insufficiency by a median time of 0.3 years (range, 0.08–10.2 years). For eight patients, both diagnoses were made at presentation. In the remaining seven patients, nephrotic syndrome developed after the PAI diagnosis by a median time of 1.9 years (range, 0.08–12.5 years), highlighting the need for surveillance of evolving disease. In 13 children where adrenal imaging was reported, 69% were noted to have adrenal calcifications (2, 3, 4, 5, 7, 9, 20, 21). In addition, five further patients had detectable adrenal calcifications on ultrasonography, although a biochemical diagnosis of adrenal failure was not provided (2, 3, 5) (Table 1). Interestingly, adrenal calcification is also a characteristic feature of lysosomal acid lipase deficiency or Wolman disease, secondary to saponification of cholesterol and fatty acid deposits within the adrenal cortex (22). The mortality rate was just under 50% for patients with PAI (50% overall for the syndrome), the majority occurring before the age of 2 years.

**Table 1:** Published SPLIS adrenal clinical phenotype (patients 1–37).

Pt no.	*SGPL1* pathogenic variant	Ethnicity	Sex	PAI presentation (age in years)	Glucocorticoid deficiency (Y/N)	Mineralocorticoid deficiency (Y/N)		Adrenal findings on imaging	Publication
1	c.7dup, p.S3Kfs*11	Spanish Roma	M	1.6	Y	U		Calcifications	Lovric (3)
2	c.7dup, p.S3Kfs*11	Spanish Roma	F	11	Y	U		U	Lovric (3)
3^†^	c.7dup, p.S3Kfs*11	Spanish Roma	F	9	Y	N		U	Prasad (4)
4^†^	c.261+1G	Peruvian	M	0.9	Y	Y		Enlarged, calcifications	Prasad (4)
5^†^	c.261+1G	Peruvian	F	0.3	Y	Y		Calcifications	Prasad (4)
6	c.395A>G, p.E132G; c.832delA, p.R278Gfs*17	French	F	2	Y	U		U	Lovric (3)
7	c.395A > G, p.E132G; c.832delA, p.R278Gfs*17	French	F	2	Y	U		U	Lovric (3)
8^†^	c.511A>G, p.N171D	Pakistani	M	0.6	Y	Y		Calcifications	Maharaj (7)
9^†^	c.518T>A, p.L173Q	Turkish	F	0.17	Y	Y		Calcifications	Menevse (20)
10	c.605C>T, p. S202L; c.1247A>G, p.Y416C	Unknown	M	2	Y	N		U	Zhao (10)
11	c.664C>T, p.R222W	Turkish	F	0.25	Y	U		U	Lovric (3)
12	c.664C>T, p.R222Q	Pakistani	M	6.5	Y	U		U	Lovric (3)
13	c.664C>T, p.R222Q	Pakistani	M	3	Y	U		U	Lovric (3)
14	c.664C>T, p.R222Q	Pakistani	M	2	Y	U		U	Lovric (3)
15^†^	c.664C>T, p.R222Q	Pakistani	M	0.6	Y	N		U	Prasad (4)
16^†^	c.664C>T, p.R222Q	Pakistani	M	<1	Y	N		U	Prasad (4)
17^†^	c.664C>T, p.R222Q	Pakistani	M	<1	Y	N		U	Prasad (4)
18^†^	c.664C>T, p.R222Q	Saudi	M	1.5	Y	N		U	Prasad (4)
19	c.665G>A, p. R222Q	Saudi	M	3	Y	N		Normal	Settas (17)
20	c.665G>A, p.R222Q	Saudi	M	6	Y	Y		U	Martin (8)
21	c.665G>A: p.R222Q	Saudi/British	M	1	Y	U		U	Alrayes (19)
22	c.868T>c, p.F290L; c.993C<G, p.Y331*	White American	M	1.2	Y		N	Calcification and enlarged	Taylor (9)
23	c.934delC, p. L312Yfs*30	White European	M	0	Y		U	Calcifications	Janecke (2)
24	c.1018C >T, p.R340W	Unknown	F	0.6	Y		Y	U	Linhares (6)
25	c.1018C >T, p.R340W	Unknown	M	0.7	Y		Y	U	Linhares (6)
26^†^	c.1018C >T, p.R340W	Turkish	M	0.4	Y		Y	Normal	Menevse (20)
27	c.1037G >T, p.S346I	Moroccan	F	NFD	U		U	Calcifications	Lovric (3)
28	c.1037G >T, p.S346I	Moroccan	F	NFD	U		U	Calcifications	Lovric (3)
29^†^	c.1049A>G, p.D350G	Turkish	F	0.75	Y		Y	Calcifications	Maharaj (21)
30	c.1077del, p.G360Afs*49; c.1058A>G, p. K353R	Unknown	M	0.1	Y		U	Calcifications	Zhao (10)
31	c.1079G>T, p.G360V	Turkish	F	0.4	Y		U	U	Saygili (18)
32	c.1233delC, p.F411Lfs*56	Afghan	M	NFD	U		U	Calcifications	Bamborschke (5)
33	c.1247A >G, p.Y416C	Mixed European	F	0.2	Y		U	U	Lovric (3)
34	c.1513C >T, p.R505*	Arabic	M	NFD	U		U	Calcifications (scan at 6 weeks of life)	Janecke (2)
35	c.1513C >T, p.R505*	Arabic	M	NFD	U		U	Calcifications (antenatal scan at 21 weeks gestation)	Janecke (2)
36	c.1566+2T>C; c.854G>A p.C285Y	Unknown	M	0.1	Y		U	Calcifications	Zhao (10)
37^†^	c.1633_1635delTTC, p.F545del	Turkish	F	0.5	Y		Y	Normal	Prasad (4)

Patients numbered according to position of *SGPL1* pathogenic variant.

^†^Patients referred to the QMUL cohort.

N, no; NFD, not formally diagnosed (however adrenal calcifications reported); PAI, primary adrenal insufficiency; U, unknown; Y, yes.

### Clinical phenotype of primary gonadal insufficiency in SPLIS

Primary gonadal failure has been reported in nine boys (Fig. 3 and Table 2), all of whom presented with bilateral cryptorchidism and seven additionally with microphallus, suggesting reduced androgen exposure or action *in utero*, most likely from the second trimester onwards. Hypospadias is not a reported clinical feature to date. Biochemical evaluations revealed raised basal gonadotrophins (LH and FSH), an exaggerated response to luteinizing hormone releasing hormone (LHRH) stimulation, impaired testosterone responsiveness to human chorionic gonadotrophin (hCG) stimulation and low anti-Müllerian hormone (AMH) levels. Inhibin B levels have not been reported in affected individuals. Six of these affected nine boys presented with biochemical PAI and hypothyroidism, although all had evidence of adrenal calcifications on imaging as well as concomitant nephrotic syndrome (Fig. 3). The majority died in early infancy; the oldest patient at the age of 12 years. To date, there have been no reports of pubertal delay in affected girls. Two girls who have survived into adolescence within our cohort, commenced menarche and have normal ovarian reserve as evidenced by sufficient AMH levels (4, 21). One has bilateral non-neoplastic ovarian calcifications identified at age 13 years, with no obvious impairment of gonadal function (21).

**Table 2:** Published SPLIS gonadal clinical phenotype, in all cases, the phenotype was noted post-birth/in early infancy.

Pt no.	*SGPL1* pathogenic variant	Ethnicity	Sex	Concomitant adrenal disease (Y/N)	Microphallus (Y/N)	Cryptorchidism (Y/N)	LH/FSH at presentation	HCG testing /LHRH and result	Anti-Müllerian hormone level	Publication
4^†^	c.261+1G	Peruvian	M	Y	Y	Bilateral	Raised (LH 36 IU/L, FSH 58 IU/L)	U	40 pmol/L (NR 600–2000, ECLIA)	Prasad (4)
8^†^	c.511A>G, p.N171D	Pakistani	M	Y	Y	Bilateral	Raised (LH 27 IU/L, FSH 71 IU/L)	No response in testosterone (1.0 nmol/L day 1, 0.9 nmol/L day 3) to hCG stimulation, ‘flat’ androstenedione and DHT response	U	Maharaj (7)
22	c.868T>c, p.F290L; c.993C<G, p.Y331*	White American	M	Y	Y	Bilateral	Raised (LH 23 IU/L, FSH 41 IU/L)	U	U	Taylor (9)
23	c.934delC, p. L312Yfs*30	White European	M	Y	Y	Bilateral	Raised*	U	U	Janecke (2)
26^†^	c.1018C >T, p.R340W	Turkish	M	Y	N	Bilateral	Raised (LH 52.6 IU/L, FSH 71.5 IU/L)	U	11.6 ng/mL (NR 14–466)	Menevse (20)
31	c.1233delC, p.F411Lfs*56	Afghan	M	Y	N	Bilateral	U	U	U	Bamborshke (5)
34	c.1513C >T, p.R505*	Arabic	M	Y	Y	Bilateral	Raised^*^	Lack of testosterone response to hCG stimulation^*^; exaggerated gonadotrophin response to LHRH	Low^*^	Janecke (2)
35	c.1513C >T, p.R505*	Arabic	M	Y	Y	Bilateral	Raised^*^	Lack of testosterone response to hCG stimulation^*^; exaggerated gonadotrophin response to LHRH	Low^*^	Janecke (2)
36	c.1566+2T>C; c.854G>A, C285Y	Unknown	M	Y	Y	U	U	U	U	Zhao (10)

Patient numbers correspond to numbers allocated for PAI phenotype in Table 1.

^*^Details of results not published;^†^Patients referred to the QMUL cohort.

FSH, follicle-stimulating hormone; hCG, human chorionic gonadotropin; LH, luteinizing hormone; LHRH, luteinizing hormone releasing hormone; N, no; U, unknown; Y, yes.

### Thyroid disease and SPLIS

Primary hypothyroidism, with mildly raised thyroid-stimulating hormone (TSH) and low free-T4, has been reported in 17 patients (Table 3) (3, 4, 5, 6, 7, 8, 9, 10, 20, 21). Goitres are not a usual feature of disease (reported in only one individual) and normal parenchyma is seen on thyroid ultrasound imaging (3, 4, 5, 6, 7, 8, 9, 10, 21). In most cases, this was identified in early infancy; however, there are reports of later diagnoses including one individual aged 12 years (4). The majority of individuals with thyroid disease (67%) have concomitant PAI and nephrotic syndrome (Fig. 3). Although mild thyroid dysfunction is reported in untreated adrenal insufficiency (23), all patients identified in this cohort required ongoing thyroxine replacement despite management of their adrenal disease. Similarly, while urinary loss of thyroid hormones and thyroid hormone binding proteins can be associated with nephrotic disease, this is generally compensated by increases in the free thyroid hormone fraction (24). SPLIS may predispose to a lower thyroid reserve with hypothyroidism occurring alongside the onset of nephrotic disease. There are also individuals, however, who have thyroid disease in the absence of nephrotic syndrome.

**Table 3:** Thyroid clinical phenotype reported in SPLIS patients.

Pt no.	*SGPL1* pathogenic variant	Ethnicity	Sex	Age of thyroid presentation (age in years)	TSH at diagnosis (mIU/L)	Free T4 at diagnosis (pmol/L)	TPO antibody status	Thyroid US findings	Concomitant adrenal disease (Y/N)	Concomitant SRNS (Y/N)	Publication
3^†^	c.7dup (p.S3Kfs*11)	Spanish Roma	F	12	12	19	U	Normal	Y	Y	Prasad (4)
4^†^	c.261+1G	Peruvian	M	1	12.9	11.6	Negative	Normal	Y	Y	Prasad (4)
5^†^	c.261+1G	Peruvian	F	0.3	8.14	13.5	Negative	Normal	Y	Y	Prasad (4)
8^†^	c.511A>G, p.N171D	Pakistani	M	0.23	25	10.7	U	Normal	Y	Y	Maharaj (7)
9^†^	c.518T>A, p.L173Q	Turkish	F	0.17	18	10.4	U	U	Y	Y	Menevse (20)
22	c.868T>C, p.F290L; c.99993C>G, p.Y331*	White American	M	0.18	13.3	18	U	Normal	Y	Y	Taylor (9)
26^†^	c.1018C >T, p.R340W	Turkish	M	0.4	9.2	12.6	U	U	Y	Y	Menevse (20)
27	c.1037G >T, p.S346I	Moroccan	F	U	U	U	U	U	Y	Y	Lovric (3)
28	c.1037G >T, p.S346I	Moroccan	F	U	U	U	U	U	Y	Y	Lovric (3)
29^†^	c.1049A>G, p.D350G	Turkish	F	11	8.2	10.5	Negative	Psammomatous calcified thyroglossal cyst, normal thyroid architecture	Y	N	Maharaj (21)
31	c.1079G>T, p.G360V	Turkish	F	0.3	U	U	U	U	Y	Y	Saygili (18)
32	c.1233delC, p.F411Lfs*56	Afghan	M	0.06	29.2	4.5	U	U	Y	Y	Bamborschke (5)
33	c.1247A >G, p.Y416C	Mixed European	F	0.17	U	U	U	U	Y	Y	Lovric (3)
36	c.1566+2T>C; c.854G>A; p.C285Y	Unknown	M	<0.1	U	U	U	U	Y	Y	Zhao (10)
37^†^	c.1633_1635delTTC, p.F545del	Turkish	F	U	20.3	13.8	Negative	Goitre	Y	Y	Prasad (4)
38	c.605C > T, p.S202L; c.946G>A, p.A316T	Mixed European	M	U	Raised^*^	U	U	U	N	Y	Lovric (3)

Patient numbers correspond to numbers allocated for PAI phenotype in Table 1.

^*^Details of results not published; ^†^Patients referred to the QMUL cohort.

N, no; T4, thyroxine; TPO, thyroid peroxidase; TSH, thyroid-stimulating hormone; U, unknown; US, ultrasound; Y, yes.

### Other endocrine manifestations of SPLIS

Given the high early childhood mortality associated with SPLIS, limited long-term growth data are available. There is no clear evidence of intrauterine growth retardation, with birthweight percentiles ranging from the 1st to the 98th centile within our cohort and further cases in the literature (2, 4, 5, 6, 7, 17). No formal assessment of the growth hormone IGF-1 axis is reported.

Two girls within our cohort have been followed up longitudinally until adult height was achieved. The first, born small for gestational age (SGA) with a birthweight SDS of −2.35, had further evidence of growth failure with a final height SDS of −3.67 (parent adjusted height SDS of −2.40) despite IGF-1 levels within normal range. She manifested PAI and hypothyroidism in the absence of renal disease. The second, with a birthweight SDS of +1.69, eventually achieved a parental adjusted height SDS of −3.02. Her clinical presentation included SRNS at age 9 months, along with PAI and hypothyroidism. She progressed through puberty normally and had received two renal transplants at ages 5 and 12. Another patient first demonstrated signs of growth restriction when he developed chronic renal failure at 10 months of age. At his last review, at age 8 years, his parental adjusted height SDS was −1.75 having previously had a birth length SDS of +0.36. His IGF-1 levels were undetectable on two separate occasions, thought to be a result of his clinical and nutritional state. Conversely, another patient, who was born SGA with a birthweight SDS of −2.64, was reviewed aged 3 years with a height SDS of +1.43. Interestingly, he harboured the p.R222Q variant and phenotypically presented with isolated adrenal disease. Thus, there is no consistent evidence of the impact of *SGPL1* on growth, both *in utero* or postnatally, and associated comorbidities may play a role.

Metabolic bone disease is also a reported feature of SPLIS and at least, three patients in our cohort were deemed to have tertiary hyperparathyroidism with a background of chronic renal disease, one necessitating parathyroidectomy.

### Genotype and correlation with endocrine phenotypes

**Figure 4:**
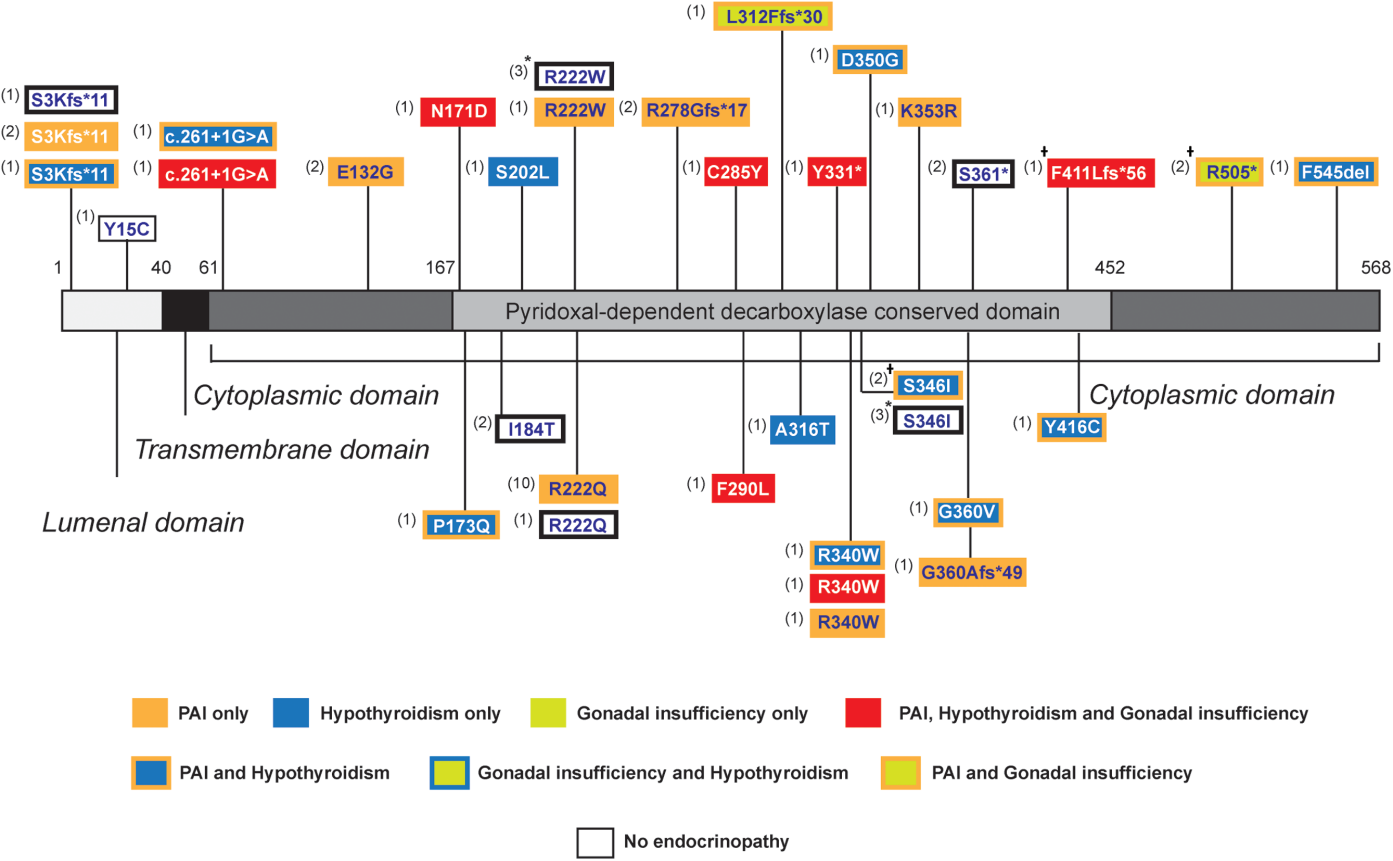
Domain topology of reported pathogenic variants in *SGPL1* and delineation of associated endocrinopathy. Numbers of patients associated with each genotype/phenotype are included in parantheses. ^†^Also includes patients with adrenal calcification where biochemical PAI has not been defined. ^*^Includes a case of fetal demise.

Bi-allelic *SGPL1* mutations include missense, frameshift and stop-gain variants, which are not ‘domain-centric’ but rather distributed across the entire gene (Fig. 4). The most prevalent genetic variant in the syndrome is the single amino acid substitution, p.R222Q, affecting 11 children from different families. This variant is almost consistently associated with glucocorticoid deficiency as the only endocrine manifestation. Mineralocorticoid deficiency is reported in only one individual with this variant and a further individual is not reported to have any endocrine phenotype. Furthermore, in five patients with PAI without associated nephrotic disease, all harboured the p.R222Q variant, with the exception of one patient with the p.D350G variant (21). Three out of five of these patients, however, had other manifestations associated with the syndrome. The p.R222Q variant has also been reported with renal disease and there is variability in overall syndrome phenotype even among kindreds (4). Aside from the relative consistency in endocrine phenotype seen with the p.R222Q variant, there are no other clear associations between endocrinopathy and genotype. Six individuals reported with all three major endocrinopathies (adrenal, gonadal and thyroid disease), all with associated nephrotic disease, had variants which were not confined to the active pyridoxal-dependent decarboxylase conserved domain of *SGPL1* but were distributed throughout the cytoplasmic domain (Fig. 4) (4, 5, 7, 9, 10, 20).

## Discussion

SPLIS has drawn attention to the role of sphingolipids in the pathogenesis of endocrine disease and the need for endocrine specialist input in the syndrome. Indeed, we are mindful that our review of the clinical phenotype is potentially limited by reporting bias within the 50 published cases and early deaths, therefore, may even underestimate the prevalence of endocrine disease. While there is no clear genotype–phenotype correlation overall in the syndrome, with regards to endocrinopathy, the most prevalent disease variant p.R222Q is associated most consistently with an isolated glucocorticoid deficiency. In the most severe cases of SPLIS, multi-endocrine dysfunction is noted in the early postnatal period. However, late manifestations also indicate scope for evolving disease. The combination of PAI and SRNS should certainly alert clinicians to the possibility of SPLIS; however, individuals can also present with isolated adrenal disease (4, 17). Mutations in *SGPL1* should therefore be considered as part of the differential diagnosis for PAI of unknown aetiology, in order to initiate early screening and appropriate treatment to reduce morbidity and mortality. This includes careful clinical review for other features of the syndrome and biochemical screening, for instance, for developing nephrotic disease (25). In the absence of concomitant nephrotic syndrome, other features including lymphopenia, ichthyosis and neurological disease may provide clues to the diagnosis (Fig. 1). Neurological disease, present in approximately half of all patients (including two PAI patients without nephrotic disease), is varied and can include microcephaly, neurodevelopmental delay and regression, sensorineural deafness, peripheral neuropathy and progressive neurological deterioration (3, 4, 5, 7, 8, 10, 16, 17, 21). Additional endocrinopathy should also raise suspicion of the syndrome (Fig. 3). Biochemical screening for endocrinopathy could involve 6–12 monthly evaluation of adrenal and thyroid function (25). A significant proportion of patients presenting primarily with nephrotic disease have had steroid treatment in the initial phase of their management which may subsequently delay the diagnosis of adrenal insufficiency. Indeed, clinicians need to be mindful of the risk of precipitating adrenal crisis with withdrawal of steroids in SPLIS in those with undiagnosed adrenal disease. We find that while there is no overall significant sex bias in syndrome presentation, there is a male preponderance among this international cohort who develop PAI. This is in keeping with findings from a UK-based PAI cohort, lending to the hypothesis that there may be sex-based differences in adrenal function rendering boys more vulnerable (26).

As a third of male patients are reported with gonadal insufficiency and, given the potential risk for progressive gonadal failure, close evaluation of puberty and adult sex steroid production is required. Consideration may be given to sperm banking for boys where appropriate, in cases of evolving disease; however, more data are required on the natural evolution of SPLIS in puberty and effects on spermatogenesis and fertility. To date, ovarian dysfunction has not been described although non-neoplastic ovarian calcifications have been reported. Unlike the fetal testis, the fetal ovary is not believed to be significantly steroidogenic, thus the ovaries may ‘escape’, whereas reduced *in utero* testicular androgen production/action from the second trimester onwards result in disorders of sex development for boys. A similar phenomenon akin to that seen in 46,XX individuals with *STAR* variants may occur, where enzyme deficiency is either compensated for, or girls progress through puberty appropriately but hypergonadotrophic hypogonadism ensues thereafter (27, 28).

The pathogenic mechanisms underlying the endocrine aspects of disease are yet to be fully determined. Adrenal and gonadal tissue from SPLIS patients have not been available to study; however, *Sgpl1*-deficient mice recapitulate the organ-specific phenotypes seen in human counterparts. *Sgpl1* ^−/−^ mice have suboptimal corticosterone production (29) and their adrenals show disrupted adrenocortical zonation, reduced expression of steroidogenic enzymes and loss of the typical sub-capsular clusters of aldosterone synthase (4). Deletion of *Sgpl1* abrogates germ cell development in gonads rendering both male and female *Sgpl1*^−/−^ mice sterile and attributed to intra-gonadal accumulation of S1P (30, 31). Testes exhibit reduced spermatids and Leydig cell numbers, while increased pre-antral follicle atresia is noted in murine ovaries (30, 31).

Trophic ACTH stimulation of NCI-H295R (adrenocortical carcinoma) cells increases S1P expression and steroidogenesis (32). Conversely, sphingolipid intermediates ceramide and sphingosine diminish steroidogenesis, with sphingosine purported to directly attenuate the activity of steroidogenic factor 1 (SF-1) (33). Despite consistent increases in S1P seen in plasma derived from patients with SGPL1 deficiency, adrenal insufficiency manifests. It is possible that on balance, a greater role is played by the upstream accumulation of other sphingolipids, ceramide and sphingosine, on steroidogenesis. S1P exerts its effects through five G protein coupled receptors (S1PR_1-5_), and its stimulation of downstream inositol triphosphate and calcium signalling can stimulate steroidogenesis (34). The increase in S1P seen in SPLIS may overwhelm S1P receptors (S1PR) resulting in their internalisation and degradation, a phenomenon that has been described in *in vitro* studies (35, 36, 37). Understanding the impact of the balance of sphingolipid intermediates, including the effect of raised S1P on downstream S1PRs, needs to be further explored in a steroidogenic model of *SGPL1* deficiency.

Patients with S1P lyase deficiency have relatively mild increases in (TSH). While sphingolipids play an important role in cell membrane integrity, the sphingolipid-cholesterol rich domains in human thyroid follicular cells do not incorporate the TSH receptor and have no effects on the TSH/ cAMP cascade thereafter (38). Ceramide does potentially reduce the transactivation capability of thyroid transcription factor 1 (*TTF-1*/*NKX2-1*) *in vitro* (39), which regulates the expression of genes essential for thyroid hormone biosynthesis (40, 41, 42, 43). While SGPL1 deficiency in humans does not consistently translate to growth failure, postnatal growth retardation is a feature seen in *Sgpl1*^−/−^ mice (30). Ongoing evaluation of auxology in all patients is required to assess any impact of SGPL1 deficiency on growth and it is possible that comorbidities including renal disease and endocrinopathy ultimately affect growth, particularly if identified late or not adequately treated.

The constellation of adrenal, gonadal and thyroid disease is described in one other non-autoimmune-inherited form of adrenal insufficiency secondary to mutations in nicotinamide nucleotide transhydrogenase (*NNT*), involved in energy transfer in the mitochondrial respiratory chain (44), although there have now also been reports of hypothyroidism adding to the phenotype of adrenal and gonadal insufficiency in MIRAGE syndrome secondary to gain of function mutations in *SAMD9* (45). Endocrine dysfunction can also be a feature of primary mitochondrial disorders associated with disruption of mitochondrial ATP production required for hormone synthesis and secretion (46). Indeed, perturbation of mitochondrial dynamics is seen in SPLIS patient dermal fibroblasts with subsequent impact on steroidogenesis (47). Mitochondrial disruption may account for the endocrine phenotype in SPLIS, setting it apart from the other disorders of sphingolipid metabolism.

There are limited reports of endocrinopathy in other sphingolipid diseases, which are in the main lysosomal storage disorders (48). Fabry disease results in systemic accumulation of globotriaosylceramide and has been associated with subclinical hypothyroidism (49, 50). In a study of 18 Fabry patients, suboptimal cortisol concentrations and higher ACTH levels when compared with controls were reported although only one patient had proven PAI after cosyntropin stimulation and one patient was diagnosed with hypergonadotrophic hypogonadism (50). Interestingly, increased S1P has been implicated in the pathophysiology of Fabry disease (51). The endocrinopathy seen in SPLIS does raise the question as to whether clinicians need to be more mindful of endocrine disease in the sphingolipidoses.

There is currently no single curative therapy for this multi-systemic disorder. Renal transplantation for the SRNS and hormone replacement for endocrine dysfunction remain the mainstay of treatment. Exogenous administration of pyridoxine (Vitamin B6), a co-factor for S1P lyase, demonstrates some improvements in immunological profiles and absolute lymphocyte counts, for patients with the p.R222Q genotype, but does not avert end organ damage or rapidity of disease progression (10). More recently, adenoviral gene transfer of human *SGPL1* to newborn *Sgpl1* null mice prolonged survival and averted development of anaemia, nephropathy, neurological compromise and lipid dyshomeostasis (29). Gene transfer did not, however, restore corticosterone production (29).

## Conclusion

Careful phenotyping of the surviving SPLIS cohort and new patients is critical in understanding the role that *SGPL1* plays in endocrine function, evolving disease and response to future treatment. Furthermore, the sphingolipid pathway is a therapeutic target in oncology and autoimmune disease (12) and further study of the effects of disrupted sphingolipid homeostasis in SPLIS will also inform on the potential endocrine impact of therapeutically targeting the pathway in other conditions.

## Declaration of interest

The authors declare that there is no conflict of interest that could be perceived as prejudicing the impartiality of this review.

## Funding

This work was supported by the Medical Research Council http://dx.doi.org/10.13039/501100000265 (MRC) UK Clinical Academic Research Partner Grant (MR/T02402X/1, 2019 to R P), Barts and the London Charity (MGU0361, 2017 to L A M), Barts and the London Charity Research Fellow Grant (MGU0528 to R K), Medical Research Council http://dx.doi.org/10.13039/501100000265 (MRC) UK Clinical Research Training Fellowship Grant (MR/W015935/1 to RK), Government of Trinidad and Tobago Research Fellowship (to A M) and Wellcome Trust http://dx.doi.org/10.13039/100010269 (209328/Z/17/Z to J C A).
